# Vitamin D Supplementation for Treating Atopic Dermatitis in Children and Adults: A Systematic Review and Meta-Analysis

**DOI:** 10.3390/nu16234128

**Published:** 2024-11-28

**Authors:** Amalie Ying Nielsen, Simon Høj, Simon Francis Thomsen, Howraman Meteran

**Affiliations:** 1 Faculty of Health and Medical Sciences, University of Copenhagen, 2200 Copenhagen, Denmark; amalie2000@gmail.com; 2 Department of Otorhinolaryngology, Head and Neck Surgery and Audiology, Rigshospitalet, 2100 Copenhagen, Denmark; simonhoej1@hotmail.com; 3 Department of Dermato-Venereology and Wound Healing Centre, Copenhagen University Hospital-Bispebjerg, 2400 Copenhagen, Denmark; simon.francis.thomsen.02@regionh.dk; 4 Department of Respiratory Medicine, Copenhagen University Hospital—Amager and Hvidovre, 2650 Hvidovre, Denmark; 5 Department of Immunology and Microbiology, University of Copenhagen, 2200 Copenhagen, Denmark; 6 Department of Respiratory Medicine, Zealand University Hospital Roskilde-Næstved, 4700 Næstved, Denmark

**Keywords:** atopic dermatitis, meta-analysis, systematic review, children, adults, efficacy, SCORAD index, human health, vitamin D

## Abstract

Background: Atopic dermatitis (AD) is a chronic inflammatory skin disease affecting up to 20% of children and 10% of adults worldwide. Current research suggests a correlation between serum vitamin D level and AD severity and that vitamin D supplementation could have a potential therapeutic effect on AD. Objectives: To conduct a systematic review and meta-analysis of studies of vitamin D supplementation for disease improvement in children and adults with AD. Methods: PubMed, EMBASE, and Cochrane were searched from 19 April to 20 April 2024. We included randomized controlled trials (RCTs) of patients with AD comparing an intervention group with a control group. The risk of bias of the selected studies was assessed using the Cochrane risk-of-bias tool for randomized trials. All analyses were conducted in R (v4.1.2; R Core Team 2021). Results: Eleven RCTs with 686 participants were included. The included trials had measured AD severity by using the SCOring Atopic Dermatitis (SCORAD) or the Eczema Area and Severity Index (EASI). Vitamin D supplementation significantly reduced AD severity compared with the control group (standardized mean difference = −0.41, 95% CI: −0.67 to −0.16, *I*^2^ = 58%, *p* < 0.01). Conclusions: Vitamin D supplementation reduces AD severity in children and adults. Larger-scale and longer-term studies are still needed to confirm this conclusion. This study has been registered on PROSPERO (CRD42024535014).

## 1. Introduction

Atopic dermatitis (AD) is a chronic inflammatory skin condition affecting up to 20% of children and 10% of adults [1,2]. AD is characterized by recurrent eczematous lesions and itching typically affecting the face, cheeks, and trunk in children and the hands, eyelids, and flexures in adults [3,4]. Onset often occurs in early childhood, with symptoms appearing in up to 45% of cases within six months and 60% within the first year of life [5].

The pathophysiology of AD is complex and involves an interplay between epidermal barrier defects, immune dysfunction, and microbiome abnormalities [3]. The skin of AD patients is characterized by an increased expression of type 2 cytokines including IL-4, IL-5, and IL-13 [6]. These cytokines are thought to play a pathogenic role in AD by suppressing antimicrobial peptides (AMPs) and thereby skin barrier functions, leading to increased susceptibility to *S. aureus* [7]. Differences in colonization of *S. aureus* and *S. epidermidis* have been demonstrated to contribute to the underlying inflammation and severity of AD [8].

AD development is associated with several risk factors such as socioeconomic status [9], weight at birth [10], and exposure to hard water [11]. Parental history of atopic diseases has been shown to be a strong risk factor [12]. Furthermore, it has been observed that loss-of-function mutations in the gene encoding the epidermal structural protein, filaggrin (FLG), are strongly associated with AD susceptibility [13]. Recent studies suggest that *FLG* mutations may have evolved as a mechanism to enhance cutaneous vitamin D synthesis in populations living at high latitudes with reduced UV-B exposure [14].

Recently, the association between vitamin D and atopic diseases has attracted attention [15]. Firstly, worsening of AD in higher-latitude countries during winter suggests a potential relationship [16]. Secondly, studies have noted improvement in AD severity with vitamin D supplementation [17]. Lastly, genetic polymorphisms in the vitamin D receptor have been linked to the pathogenesis of AD [18].

Vitamin D is known to regulate both immune responses and skin barrier function. Early studies indicate that vitamin D boosts innate immunity by enhancing antimicrobial effects of macrophages and monocytes [19]. Recent research highlights its crucial role in wound healing [20]. Additionally, studies suggest that vitamin D promotes synthesis of filaggrin, improving the skin barrier and the expression of antimicrobial peptides (AMPs) including cathelicidin, thus preventing skin infections [21].

Given its potential to support skin barrier integrity, enhance antimicrobial peptide activity, and suppress inflammation, vitamin D supplementation emerges as a possible therapeutic option for AD. However, studies have reported conflicting results [22,23], and although multiple studies have shown associations between low serum vitamin D levels and severity of AD, the correlation may not be causal. We conducted a systematic review and meta-analysis of randomized controlled trials to determine the impact of vitamin D supplementation on AD disease severity.

## 2. Materials and Methods

This systematic review was performed in accordance with the Preferred Reporting Items for Systematic reviews and Meta-Analyses (PRISMA) statement 2020 [24] and has been registered on PROSPERO (CRD42024535014).

### 2.1. Search Method

We conducted our searches from the databases PubMed, EMBASE, and the Cochrane Central Register of Controlled Trials from 19 April to 20 April 2024. The search was performed using the following keywords: (atopic dermatitis OR eczema OR dermatit*) AND (vitamin D OR vitamin D3 or cholecalciferol) AND (supplementation OR treatment OR therap*). The search was limited to human studies published in English, Danish, Swedish, or Norwegian. We did not impose any restrictions on period or article type. In addition, we manually checked reference lists of relevant articles.

### 2.2. Type of Studies and Outcomes

We included randomized controlled trials (RCTs). Studies must have included patients with a clinical diagnosis of AD. We did not impose restrictions regarding age, sex, nationality, or any other demographic factors. Studies must have assessed the use and effects of vitamin D supplementation as the only intervention. Studies that reported vitamin D supplementation combined with other treatments were excluded. We did not impose restrictions regarding type of vitamin D, route of administration, dose of vitamin D, frequency, or duration of treatment.

Main outcomes were determined by change in severity of AD measured by the following validated clinical tools: SCOring Atopic Dermatitis (SCORAD), Eczema Area and Severity Index (EASI), Physician Global Assess (PGA), Body Surface Area (BSA), Atopic Dermatitis Severity Index (ADSI), Patient Oriented Eczema Measure (POEM), Dermatology Life Quality Index (DLQI), and Pruritus Numerical Rating Scale (NRS). Additionally, number of AD-related health care visits and flare-ups were accepted as measures of severity change. Studies that lacked sufficient data on outcome measures were excluded from the meta-analysis but kept in the qualitative review.

### 2.3. Study Selection and Data Extraction

**Figure 1:**
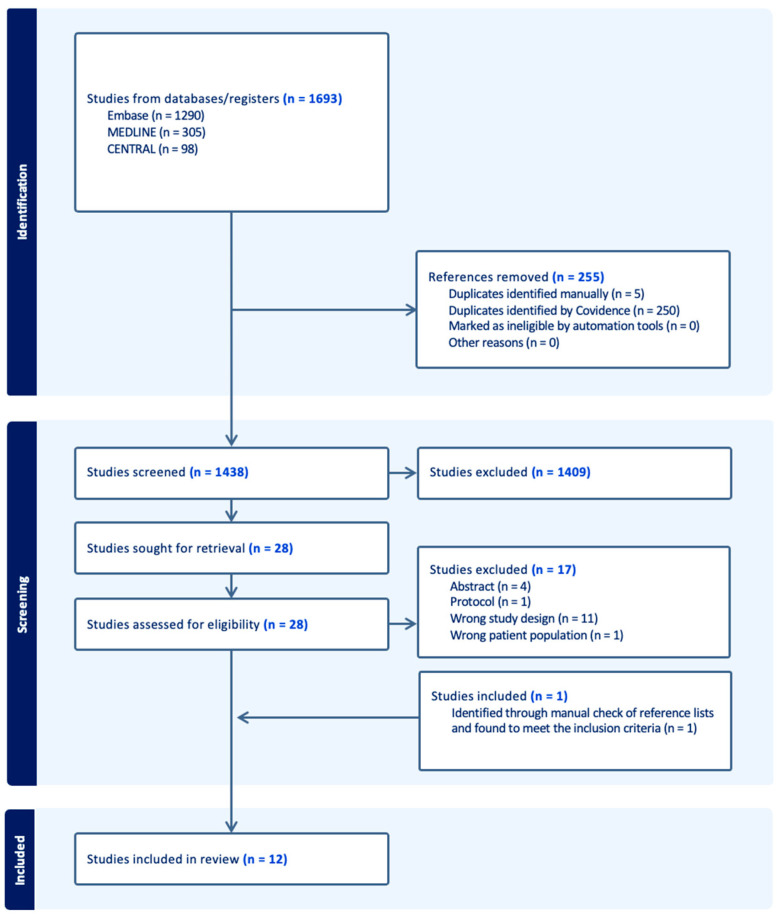
PRISMA flowchart of literature screening and selection. “n” is the number of studies.

Two authors (A.Y.N. and H.M.) independently screened the titles and abstracts obtained from the literature search. Following this, both authors independently reviewed the full text of selected articles to determine final eligibility. Any disagreements were resolved through discussion between all authors. One author (A.Y.N.) read all eligible articles in detail and extracted the data using a standard data extraction sheet, while the others checked for accuracy. The following data were extracted: the name of first author, year of publication, country, type of study, size of study population, study population characteristics, baseline values, route of administration, frequency of supplementation, dose of vitamin D, duration of intervention, outcome(s), and evaluation of the risk of bias. Studies that lacked sufficient quantitative data on outcome measures were excluded from the meta-analysis but kept in the review. A flowchart of the selection process is shown in Figure 1. Further details of the search strategies can be found in Table A1 in Appendix A.

### 2.4. Quality Assessment

Two authors (A.Y.N. and S.H.) independently assessed the risk of bias (RoB) of the selected studies using the Cochrane risk-of-bias tool for randomized trials (RoB 2) [25]. Two authors (A.Y.N. and S.H.) evaluated the overall quality of evidence with the Grading of Recommendations Assessment, Development, and Evaluation (GRADE) approach.

### 2.5. Statistical Analysis

A random-effects model was employed to calculate the pooled standardized mean difference (SMD), accounting for both within-study and between-study variability. Heterogeneity was assessed using the *I*^2^ and Tau^2^ statistics.

To ensure the integrity of the findings, an influence analysis was performed to examine the impact of individual studies on the overall meta-analysis estimate. This included a leave-one-out analysis as well as the evaluation of rstudent values, DFFITS values, and Cook’s distances. Baujat diagnostics were also employed to assess the contribution of individual studies to overall heterogeneity.

Potential publication bias was evaluated through funnel plot analysis for asymmetry, with Egger’s test applied for further confirmation. Sensitivity analysis, including outlier detection, was conducted to ensure the robustness of the meta-analytic results. All statistical analyses were performed using R (v4.1.2; R Core Team, R Foundation for Statistical Computing, Vienna, Austria, 2021), with results presented as SMDs and corresponding 95% confidence intervals. Statistical significance was defined as a *p*-value less than 0.05.

### 2.6. Subgroup Determination

For the subgroup analysis, the following subgroups were included: duration of intervention, baseline severity of AD, and vitamin D dose. For the duration of intervention analysis, participants were divided into two groups (<3 months and ≥3 months). For the baseline severity of AD analysis, participants were divided into two groups (mild-to-moderate and severe). For the vitamin D dose analysis, participants were divided into two groups (<1000 IU/day and ≥1000 IU/day).

## 3. Results

The search yielded 1438 articles after duplicates were removed. The title and abstract screening by two authors resulted in the selection of 28 articles for full-text review. The full-text review by two authors resulted in the inclusion of 10 studies. Hata et al. [26] was excluded due to lack of data. Additionally, we performed a citation search, identifying one additional study that met the inclusion criteria [27], resulting in the inclusion of 11 RCTs in the qualitative review and meta-analysis.

### 3.1. Characteristics of Studies

The 11 articles included in this review all studied the effects of vitamin D supplementation on AD severity. The sample sizes ranged from 11 to 104. Publication dates ranged from 2008 to 2024. While all studies administered vitamin D supplementation orally, there was variability in the type, dosage, frequency, and duration of supplementation. Dosages ranged from 1000 to 5000 international units (IUs) per day and 8000 to 60,000 IUs per week, with treatment durations spanning 4 to 12 weeks. Cholecalciferol was predominantly used, except for one study which utilized ergocalciferol [17]. The 11 studies had a total of 686 participants of which 51% were male. Most of the studies were conducted with children, but two studies included adults as well [28,29]. The average age was 12 years. Table 1, Table 2 and Table 3 show the characteristics and baseline values of each included RCT for the total study group, the intervention group, and the control group.

### 3.2. Risk of Bias in Studies

**Figure 2:**
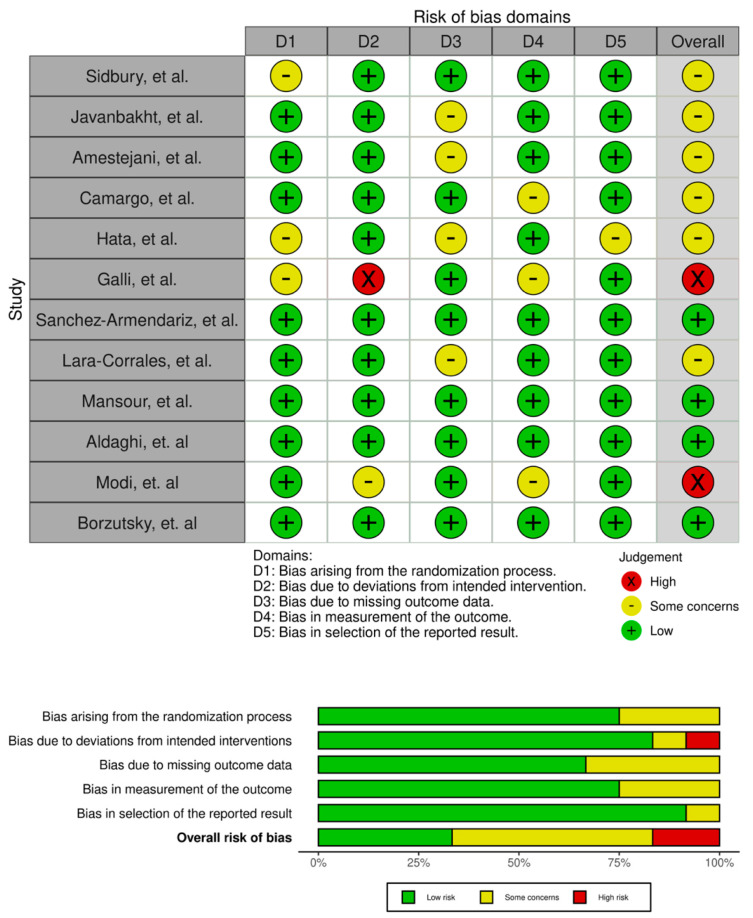
Risk assessment of bias in RCTs [17,26,27,28,29,30,31,32,33,34,35,36].

Two RCTs did not report any placebo treatment in the control group and thus did not blind patients and researchers, leading to high risk of bias in the blinding domain [31,36]. Two studies had unclear risk of bias regarding the randomization process [17,31]. Four RCTs had low risk of bias in all domains [27,32,34,35], whereas the rest had unclear risk of bias in at least one domain. Figure 2 shows an overview of the RoB assessment for each RCT.

### 3.3. Effect of Vitamin D Supplementation on AD Severity

Assessment of AD severity relied on the SCORAD index in seven studies, while four trials employed the EASI score [17,27,30,34]. Notably, no adverse effects related to the treatment were reported in any of the studies. Additionally, a subset of studies conducted sample size calculations, which were all deemed adequate [27,30,31,32,34,35]. Table 3 shows a summary of the results.

### 3.4. Meta-Analysis

**Figure 3:**
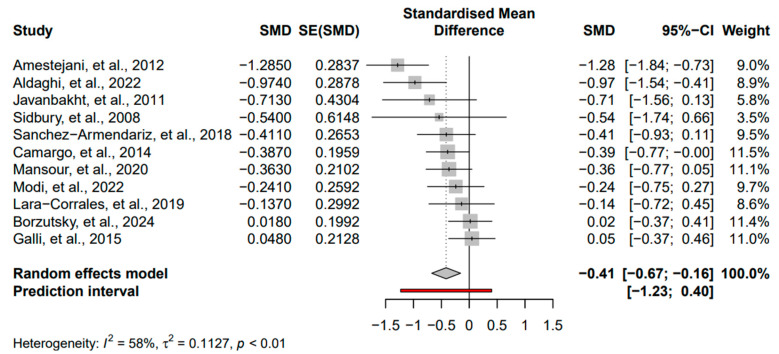
Forest plot for meta-analysis of the effect of vitamin D intervention on AD severity [17,27,28,29,30,31,32,33,34,35,36]. Square: Individual study estimates; Rhombus: Pooled effect estimate; Red: Prediction interval.

**Figure A1:**
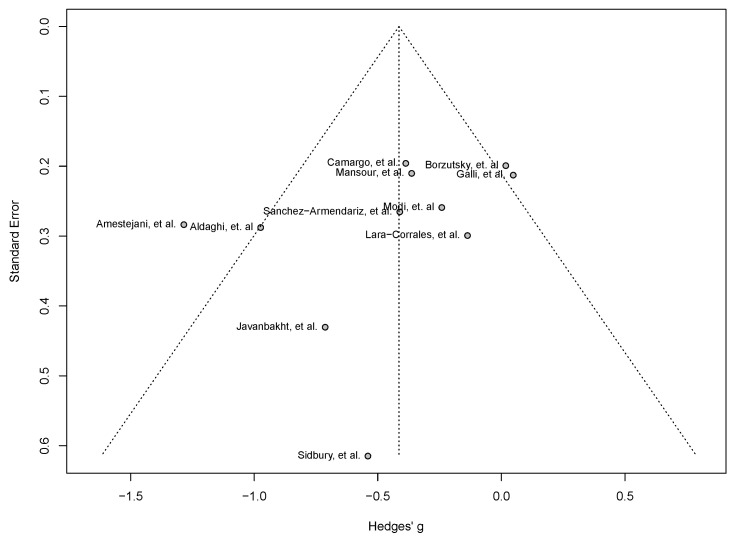
Comparative meta-analysis funnel chart [17,27,28,29,30,31,32,33,34,35,36].

**Figure A2:**
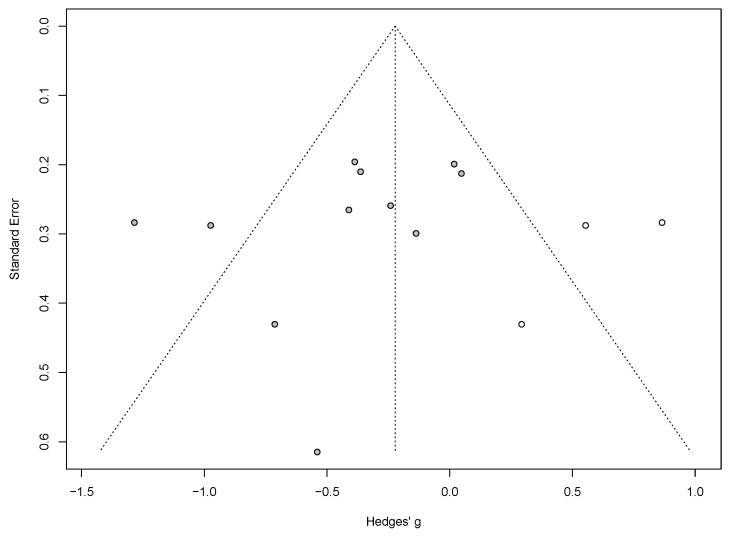
Trim-filled funnel chart [17,27,28,29,30,31,32,33,34,35,36].

Eleven studies with a total of 686 participants reported the primary outcome and were included in the meta-analysis. Significant improvement in AD symptoms following vitamin D supplementation was found in 27% (3/11) of studies [29,30,35]. The pooled results found that vitamin D supplementation significantly reduced AD severity in the intervention group compared with the control group (SMD = −0.41, 95% CI: −0.67 to −0.16, *I*^2^ = 58%, *p* < 0.01) (Figure 3). Egger’s test did not indicate the presence of funnel plot asymmetry, thus supporting the absence of publication bias (Figure A1) and (Figure A2). Using the GRADE approach, the quality of this evidence was downgraded to moderate due to uncertain risk of bias and imprecision.

### 3.5. Outlier Detection and Influence Analysis

**Figure A3:**
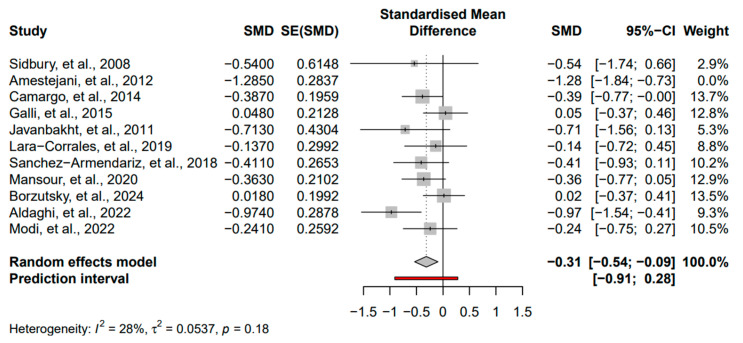
Outlier detection [17,27,28,29,30,31,32,33,34,35,36]. Square: Individual study estimates; Rhombus: Pooled effect estimate; Red: Prediction interval.

**Figure A4:**
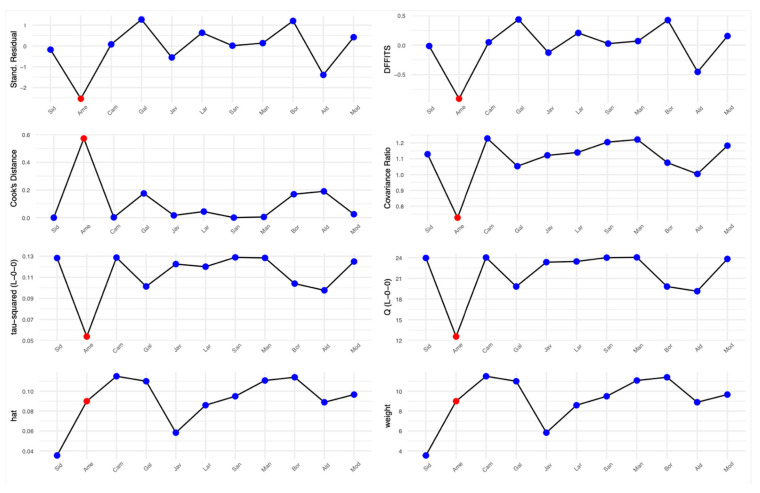
Influence statistics. Red: Outliers or critical values; Blue: Values within the acceptable range.

To assess the potential impact of outliers on the overall meta-analytic model, we conducted an outlier detection analysis. The study by Amestejani, et al. was identified as a significant outlier. Upon removal of this outlier, the random-effects model yielded a standardized mean difference (SMD) of −0.315 (95% CI: −0.535 to −0.094), indicating a statistically significant moderate effect size (*p* = 0.005). The prediction interval ranged from −0.909 to 0.280, suggesting potential variation in the effect sizes of future studies, with estimates spanning from moderate negative to small positive effects. Heterogeneity was moderately reduced after the removal of the outlier, with *I*^2^ = 28.4% and τ^2^ = 0.0537. The test for heterogeneity was not significant (Q = 12.56, *p* = 0.183), indicating that the residual between-study variance was within an acceptable range (Figure A3 and Figure A4).

**Figure A5:**
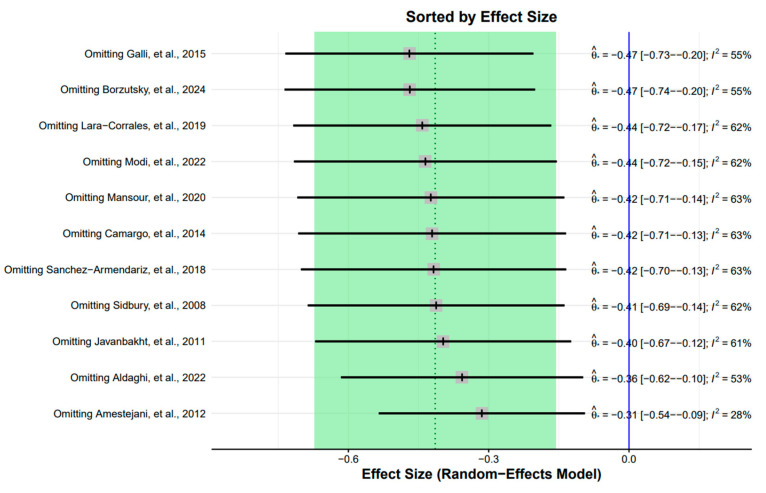
Leave-one-out analysis [17,27,28,29,30,31,32,33,34,35,36].

To further explore the impact of individual studies, we performed a leave-one-out influence analysis. This analysis revealed that omitting Amestejani, et al. resulted in minimal changes to the overall effect size (SMD = −0.315, 95% CI: −0.535 to −0.094, *I*^2^ = 28.4%), confirming that the exclusion of this study did not substantially alter the conclusions of the meta-analysis. However, additional studies such as Aldaghi, et al. [35], Galli, et al. [31] and Borzutsky, et al. [27] exhibited moderate influence on both the overall effect size and the heterogeneity, with their exclusion leading to higher heterogeneity (*I*^2^ ranging from 53.0% to 62.6%) (Figure A5).

**Figure A6:**
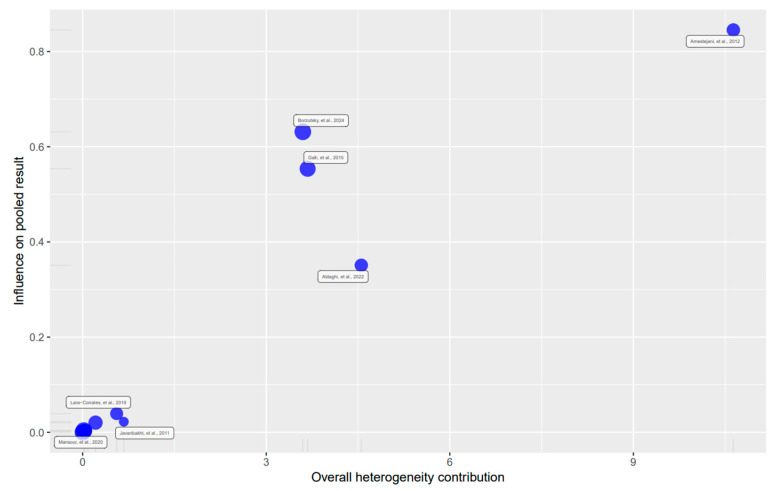
Baujat diagnostics [17,27,28,29,30,31,32,33,34,35,36].

Finally, Baujat diagnostics indicated that Amestejani, et al. [29] contributed the most to heterogeneity (HetContrib = 10.634) and had the largest influence on the effect size (InfluenceEffectSize = 0.845). Other studies, including Aldaghi, et al. [35] and Galli, et al. [31] also contributed meaningfully to heterogeneity, though to a lesser extent. These results suggest that while certain studies exert some influence on the overall model, the primary outlier was Amestejani, et al., and its removal substantially improved model stability and precision (Figure A6).

### 3.6. Subgroup Analysis

**Figure 4:**
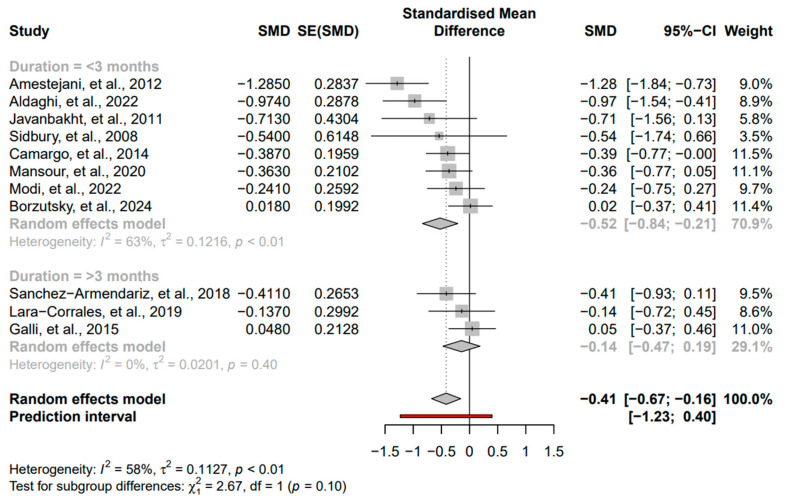
Subgroup analysis for duration based on the groups <3 months and ≥3 months [17,27,28,29,30,31,32,33,34,35,36]. Square: Individual study estimates; Rhombus: Pooled effect estimate; Red: Prediction interval.

**Figure 5:**
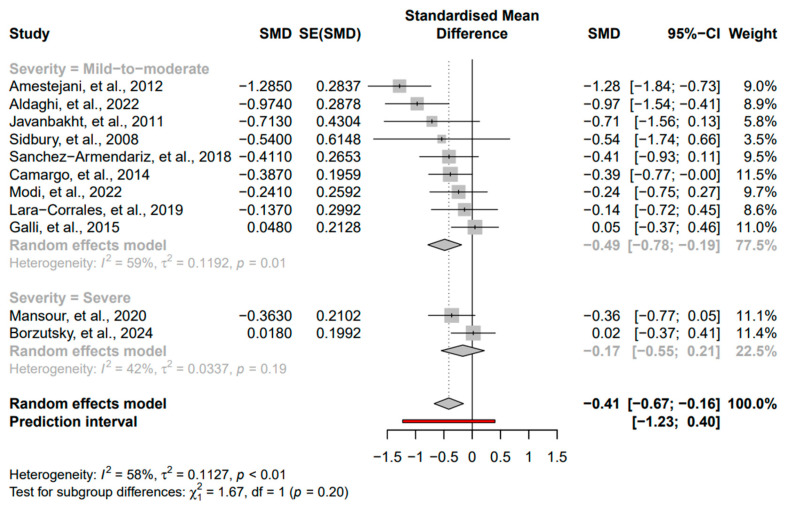
Subgroup analysis for severity based on the groups mild-to-moderate and severe [17,27,28,29,30,31,32,33,34,35,36]. Square: Individual study estimates; Rhombus: Pooled effect estimate; Red: Prediction interval.

**Figure 6:**
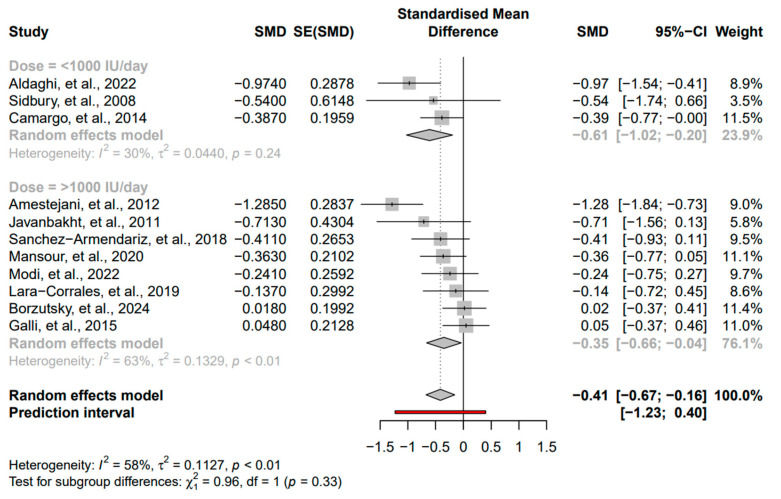
Subgroup analysis for dose based on the groups >1000 IU/day and <1000 IU/day [17,27,28,29,30,31,32,33,34,35,36]. Square: Individual study estimates; Rhombus: Pooled effect estimate; Red: Prediction interval.

The following subgroups were evaluated: duration of intervention, baseline severity of AD, and vitamin D dose. For the duration analysis on the two groups (<3 months and ≥3 months), we found a stronger and significant effect in the <3 months group (SMD = −0.52, 95% CI: −0.84 to −0.21, *I*^2^ = 63%, *p* < 0.01) compared to a non-significant effect in the ≥3 months group (SMD = −0.14, 95% CI: −0.47 to 0.19, *I*^2^ = 0%, *p* = 0.4) (Figure 4). For the severity analysis on the two groups (mild-to-moderate and severe), we found a stronger and significant effect in the mild-to-moderate group (SMD = −0.49, 95% CI: −0.78 to −0.19, *I*^2^ = 59%, *p* = 0.01) compared to a non-significant effect in the severe group (SMD = −0.17, 95% CI: −0.55 to 0.21, *I*^2^ = 42%, *p* = 0.19) (Figure 5). For the vitamin D dose analysis on the two groups (>1000 IU/day and <1000 IU/day), we found a significant effect in the >1000 IU/day group (SMD = −0.35, 95% CI: −0.66 to −0.04, *I*^2^ = 63%, *p* < 0.01) compared to a non-significant effect in the <1000 IU/day group (SMD = −0.41, 95% CI: −1.02 to −0.20, *I*^2^ = 30%, *p* = 0.24) (Figure 6).

## 4. Discussion

This systematic review and meta-analysis found that vitamin D supplementation significantly reduced the severity of AD.

According to our knowledge, our study is the most comprehensive and up-to-date meta-analysis to assess the efficacy of vitamin D supplementation on the severity of AD in both children and adults, thus increasing the statistical power and providing a broader picture of the potential of vitamin D. Several systematic reviews have found that AD patients have a lower serum vitamin D level as compared with healthy controls and that lower serum vitamin D is associated with more severe AD [23,37]. Given that vitamin D is vital for bolstering the skin barrier defense, a diminished baseline level of vitamin D may predispose individuals to develop AD. Vitamin D helps maintain the lipid barrier, ensuring skin hydration and facilitating AMP production in the skin by regulating glucosylceramides [38]. Notably, an AD diagnosis might influence behavioral changes, potentially altering sun exposure or dietary habits, which could lead to changes in vitamin D levels.

A causal explanation for serum vitamin D levels and AD remains elusive. Several hypotheses have been proposed to explain how vitamin D could affect the severity of AD. Firstly, research has suggested that vitamin D’s preserving effect on the skin barrier could reduce secondary infections worsening AD symptoms. Secondly, vitamin D may alleviate chronic inflammation in the skin [39]. One of the included studies found a dose-response link between vitamin D levels and AD, noting stronger effect modification in vitamin D-deficient rather than non-deficient AD patients [40].

The effect of vitamin D supplementation on AD severity varied among RCTs. The studies assessed the effect using the SCORAD or EASI score. Using different methods in terms of measuring outcomes can account for inconsistent results due to differences in criteria, scoring range, and emphasis on certain aspects of AD. The SCORAD score ranges from 0 to 103, incorporating both clinical signs and subjective symptoms, whereas the EASI score ranges from 0 to 72 and is entirely based on clinical observations, making it more objective. One study suggests that patients with localized moderate-to-severe lesions have higher SCORAD but low EASI [41], the difference being that EASI does not score xerosis or oozing. On the contrary, inclusion of these in SCORAD could potentially alter its responsiveness, since xerosis can be present without active AD lesions. Our study mainly included mild-to-moderate cases. We used a random-effects model which can manage inconsistency to some extent. However, the pooled results are likely still influenced, especially since SCORAD was used in 7 out of 11 studies, thus more frequently than EASI.

Treating AD often requires multiple different approaches. First-line treatments typically focus on reducing inflammation, restoring skin barrier, and alleviating itching. These include topical corticosteroids and topical calcineurin inhibitors for mild-to-moderate cases and systemic corticosteroids, cyclosporine, methotrexate, and biologics, such as Dupilumap, for severe cases. Our study aimed to investigate the role of vitamin D isolated from other AD treatments. Current research on vitamin D supplements as an adjuvant therapy underlines its potential to improve clinical outcomes for AD patients [32,34]. If given in the right doses, vitamin D supplements are safe and may offer additional benefits in terms of immune support and skin health [42]. Still, more studies on the effects of vitamin D supplementation in conjunction with other AD treatments are needed.

The clinical implications for using vitamin D supplements in AD patients are numerous. One important implication is the role of vitamin D in immune modulation, inflammation control, and skin health. Supplementation should especially be considered in patients with vitamin D deficiency since these patients could particularly benefit from it [40]. AD tends to worsen during winter, possibly due to reduced sunlight exposure and dryer conditions [17]. Supplementation during winter may help mitigate seasonal flare-ups by compensating for lower sun exposure and reduced endogenous vitamin D synthesis. Lastly, children, individuals with higher skin melanin content, individuals living in northern latitudes, obese patients, and patients with fat malabsorption are at greater risk of vitamin D deficiency and may likewise benefit from vitamin D supplementation. Pediatric patients especially should be considered, since early control of AD may reduce risk of progression to other atopic diseases known as the “atopic march” [43].

There is currently no consensus about optimal baseline levels, dosage, or duration of vitamin D supplementation. The National Institutes of Health guidelines recommend daily upper limits of 1000–3000 IU for children up to 8 years and 4000 IU for those older than 8 years [44]. High levels of vitamin D can be harmful and cause vomiting, muscle weakness, kidney stones, and, in extreme cases, kidney failure and cardiac arrythmia. Sanchez-Armendariz et al. administered a daily dosage of 5000 IU, Modi et al. a weekly dosage of 60,000 IU, and Borzutsky et al. a weekly dosage of 8000–16,000 IU. All three trials were performed on children.

The optimal age for initiating vitamin D supplementation remains uncertain. Javanbakht et al. and Amestejani et al. focused on adult subjects, whereas the rest focused on children. A recent RCT found that antenatal supplementation had a prophylactical effect on the risk of infant AD [45]. Additionally, the appropriate stage of AD for initiating vitamin D supplementation needs elucidation. One study observed that high-dose vitamin D supplementation during infancy correlated with higher prevalence of atopic diseases, suggesting that vitamin D supplementation should be provided carefully [46].

Recent studies use Mendelian Randomization (MR) to investigate the causal link between vitamin D and AD. These studies analyze the causal association between single nucleotide polymorphisms related to vitamin D levels and AD. Two studies found low evidence that vitamin D levels causally affect AD risk, suggesting that lifestyle factors like obesity and physical inactivity may confound the correlation between vitamin D and AD [47,48]. Moreover, Drodge et al. (2021) showed that AD increases vitamin D levels, even after adjusting for vitamin D supplementation, possibly due to behavioral changes. Excluding the *FLG* locus, strongly linked to AD, still revealed evidence of a causal effect on vitamin D. The UVB-VD hypothesis suggests that transurocanic acid, an FLG breakdown product, protects the epidermis against UVB, which may increase vitamin D synthesis in individuals with *FLG*-null mutations [49]. This mechanism is believed to confer benefits in northern latitudes, potentially explaining the latitude-dependent variations in *FLG* mutation frequency observed [50].

Future epidemiological, clinical, and basic immunological studies should aim to include larger sample sizes, improving statistical power seeking to determine whether vitamin D is an effective therapy in AD. Long-term RCTs with well-defined regimens have the potential to enable differentiation between distinct categories such as age, latitude, baseline vitamin D and severity level, genetic predisposition, etc. A recent meta-analysis accounting for heterogeneities found a significant difference in the therapeutic effect of vitamin D supplementation across different age groups, geographic locations, and vitamin D dosage levels [51]. Thus, interventional trials with vitamin D dosage titration based on specific patient types and phenotypes are needed to optimize and personalize vitamin D treatment of AD. Trials comparing vitamin D supplementation in isolation and in combination with other treatment options such as phototherapy and topical corticosteroids could evaluate the medical necessity or verify potential synergistic effects. Studies designed to investigate the link between vitamin D and immunological changes in AD patients are essential to advance our understanding of the pathophysiology of AD. 

The promising efficacy of vitamin D, coupled with its low occurrence of side effects and low economic costs compared to alternative treatments, underscores its clinical relevance.

### Limitations

Our study has several limitations. Firstly, given the heterogeneity of participants, confounding factors such as latitude, dietary habits, sun exposure, obesity, and level of activity could have influenced our results. It is important to note that the trials in our analysis mainly included mild and moderate cases of AD, lacking data on the effect of vitamin D supplementation on severe cases of AD. We did not include data from pregnant women. Differences in baseline vitamin D levels varied dramatically, ranging from 9.1 to 56 ng/mL, which could be indications of problems with the randomization process or underlying confounding factors. Neither Galli et al. nor Modi et al. provided a placebo to the control group; thus, they did not blind the participants, and they received high risk in RoB [31,36]. Lastly, the total number of RCTs was small, and 10 out of 12 had sample sizes with *n* < 100.

## 5. Conclusions

This systematic review and meta-analysis found that vitamin D supplementation reduced the severity of AD in children and adults, suggesting that vitamin D treatment can be considered a safe therapeutic option. Large-scale, well-designed RCTs are needed to determine the best vitamin D regimen and define the patient type who would benefit most from vitamin D supplementation.

## Figures and Tables

**Table 1:** Summary of the characteristics of the total study group of the included RCTs.

Author	Year	Country	*n*	Route	Dose of Vitamin D, IU	Frequency	Duration, Days	Outcome Measure
Sidbury et al. [17]	2008	Britain	11	Oral	1000	1/day	30	EASI
Javanbakht et al. [28]	2011	Iran	23	Oral	1600	1/day	60	SCORAD
Amestejani et al. [29]	2012	Iran	55	Oral	1600	1/day	60	SCORAD
Camargo et al. [30]	2014	Mongolia	104	Oral	1000	1/day	30	EASI
Galli et al. [31]	2015	Italy	89	Oral	2000	1/day	90	SCORAD
Sanchez-Armendariz et al. [32]	2018	Mexico	58	Oral	5000	1/day	90	SCORAD
Lara-Corrales et al. [33]	2019	Canada	45	Oral	2000	1/day	90	SCORAD
Mansour et al. [34]	2020	Egypt	86	Oral	1600	1/day	84	EASI
Aldaghi et al. [35]	2022	Iran	54	Oral	1000	1/day	56	SCORAD
Modi et al. [36]	2022	India	60	N/A	60,000	1/week	42	SCORAD
Borzutsky et al. [27]	2024	Chile	101	Oral	8000–16,000	1/week	42	EASI

**Table 2:** Summary of the characteristics of the intervention group of the included RCTs. N/A is not applicable.

	Intervention Group					Control Group				
Author	*n*	Age, yr	Sex, %Male	Baseline Severity Score	Baseline Vitamin D, ng/mL	*n*	Age, yr	Sex, %Male	Baseline Severity Score	Baseline Vitamin D, ng/mL
Sidbury [17]	5	N/A	N/A	N/A	N/A	6	N/A	N/A	N/A	N/A
Javanbakht [28]	11	21	33	36	N/A	12	26	10	31.7	N/A
Amestejani [29]	29	N/A	N/A	24.8	9.1	24	N/A	N/A	25.3	10.2
Camargo [30]	57	9	60	21	N/A	47	9	58	21	N/A
Galli [31]	41	7.6	22	12.2	56	48	4.8	26	22.1	41.6
Sanchez-Armendariz [32]	29	12.9	N/A	41.3	21.3	29	12.2	N/A	39.8	21.6
Lara-Corrales [33]	21	8.1	48	27.3	18.1	24	8.5	58	24.4	16.2
Mansour [34]	44	12	59	44.4	22.8	42	11	43	46.4	25.4
Aldaghi [35]	27	0.37	52	34.4	N/A	27	0.51	52	30.1	N/A
Modi [36]	30	7.4	57	47.8	17.6	30	7.7	53	49.2	17.3
Borzutsky [27]	53	5.8	53	32.6	18.1	48	6.9	52	17.7	17.7

**Table 3:** Summary of the results of the included RCTs. N/A is not applicable.

	Intervention Group		Control Group		Outcome	
Author	Final Severity Score	Final Vitamin D Score	Final Severity Score	Final Vitamin D Score	Difference	Outcome Measure
Sidbury [17]	N/A	N/A	N/A	N/A	−2.4	EASI
Javanbakht [28]	23.3	N/A	22.3	N/A	−3.3	SCORAD
Amestejani [29]	15.3	22.15	23.45	9.8	−7.66	SCORAD
Camargo [30]	14.5	N/A	17.7	N/A	−3.2	EASI
Galli [31]	12	105.9	20.8	42	1.1	SCORAD
Sanchez-Armendariz [32]	20.1	58.5	25.9	22.2	−7.3	SCORAD
Lara-Corrales [33]	15.4	31.3	15.1	15.8	−2.8	SCORAD
Mansour [34]	20.42	36.11	27.47	25.86	−5.05	EASI
Aldaghi [35]	N/A	N/A	N/A	N/A	−12.38	SCORAD
Modi [36]	3.6	N/A	7.3	N/A	−2.3	SCORAD
Borzutsky [27]	27.3	34.6	26.8	18.5	−0.28	EASI

## Data Availability

The data presented in this study are available on request from the corresponding author. The data are not publicly available due to instutional policies.

## Appendix A

**Table A1:** Literature search strings.

Search Date	Database	Search Strategy
19 April 2024	PubMed (Medline)	1: “Dermatitis, Atopic”[Mesh] OR “Eczema”[Mesh] OR “Dermatitis, atopic”[Title/Abstract] OR “Eczema”[Title/Abstract] OR “Dermatit*”[Title/Abstract] 2: “Vitamin D”[MeSH] OR “Vitamin D3 24-Hydroxylase”[MeSH] OR “Cholecalciferol”[MeSH] OR “Vitamin D”[Title/Abstract] OR “Vitamin D3 24-Hydroxylase”[Title/Abstract] OR “Cholecalciferol”[Title/Abstract] OR “Vitamin D3”[Title/Abstract] 3: “Therapy” [Subheading] OR “Therapeutics”[Mesh] OR “Treatment Outcome”[Mesh] OR “Therapy”[Title/Abstract] OR “Therapeutics”[Title/Abstract] OR “Treatment Outcome”[Title/Abstract] OR “Treatment Effect Heterogeneity”[Title/Abstract] OR “Supplementation”[Title/Abstract] 4: 1 AND 2 AND 3 NOT
19 April 2024	Cochrane	#1) (MeSH descriptor: [Dermatitis, Atopic]) OR “atopic dermatitis” OR “atopic dermatit” OR “eczema” OR “Dermatit” #2) (MeSH descriptor: [Cholecalciferol]) OR “cholecalciferol” OR “vitamin D” OR “vitamin D 24-hydroxylase” OR “vitamin D-24-hydroxylase” OR “vitamin D(3)” OR “vitamin D-3” #3) “treat*” OR “therap*” OR “supplementation*” #4) #1 AND #2 AND #3
20 April 2024	Embase	1: (Exp atopic dermatitis/or exp infantile eczema/) OR (atopic dermatitis OR infantile eczema OR eczema OR dermatit).ti,ab,kf. 2: (Exp vitamin d/) OR (vitamin D OR vitamin D 24-hydroxylase OR vitamin D-24-hydroxylase OR vitamin D3 OR vitamin D 3 OR vitamin D-3).ti,ab,kf. 3: (exp treatment outcome/) OR (treat* or therap* or supplementation*).ti,ab,kf. 4: 1 AND 2 AND 3
